# Complement in Antibody-Mediated Rejection of the Kidney Graft: From Pathophysiology to Clinical Practice

**DOI:** 10.3390/jcm14082810

**Published:** 2025-04-18

**Authors:** Bogdan Marian Sorohan, Dorina Tacu, Constantin Gîngu, Silviu Guler-Margaritis, Bogdan Obrișcă, Maria-Daniela Tănăsescu, Gener Ismail, Cătălin Baston

**Affiliations:** 1 Carol Davila University of Medicine and Pharmacy, 050474 Bucharest, Romania; constantin.gingu@umfcd.ro (C.G.); silviu.guler-margaritis@umfcd.ro (S.G.-M.); bogdan.obrisca@umfcd.ro (B.O.); maria.tanasescu@umfcd.ro (M.-D.T.); gener.ismail@umfcd.ro (G.I.); catalin.baston@umfcd.ro (C.B.); 2 Department of Kidney Transplantation, Fundeni Clinical Institute, 022328 Bucharest, Romania; ticu_dorina@yahoo.com; 3 Department of Nephrology, Fundeni Clinical Institute, 022328 Bucharest, Romania; 4 Department of Nephrology, Emergency University Hospital, 022328 Bucharest, Romania

**Keywords:** kidney, transplant, antibody, rejection, complement, pathophysiology, treatment

## Abstract

Antibody-mediated rejection (AMR) is a leading cause of kidney graft failure. Complement activation is involved in the AMR process. Our aim is to provide the current understanding of the pathophysiology related to complement-mediated injury in AMR, to present the current evidence regarding complement blockade in AMR management, and to point out emerging therapies and future directions in this area. The complement system plays an important role in the onset and progression of AMR. There is a balance between complement-dependent and -independent mechanisms in the development of rejection lesions. Classic and leptin pathways are involved in this process. C4d positivity is no longer a mandatory feature for AMR diagnosis but remains an independent predictor of negative outcomes. The current evidence regarding AMR treatment is limited. Terminal and proximal complement blockade has gained recognition in clinical practice. Eculizumab and C1 inhibitors are effective in the treatment of AMR as adjuvant therapies to the standard of care. The availability of novel complement inhibitors will lead to more effective and tailored treatment strategies.

## 1. Introduction

**Figure 1:**
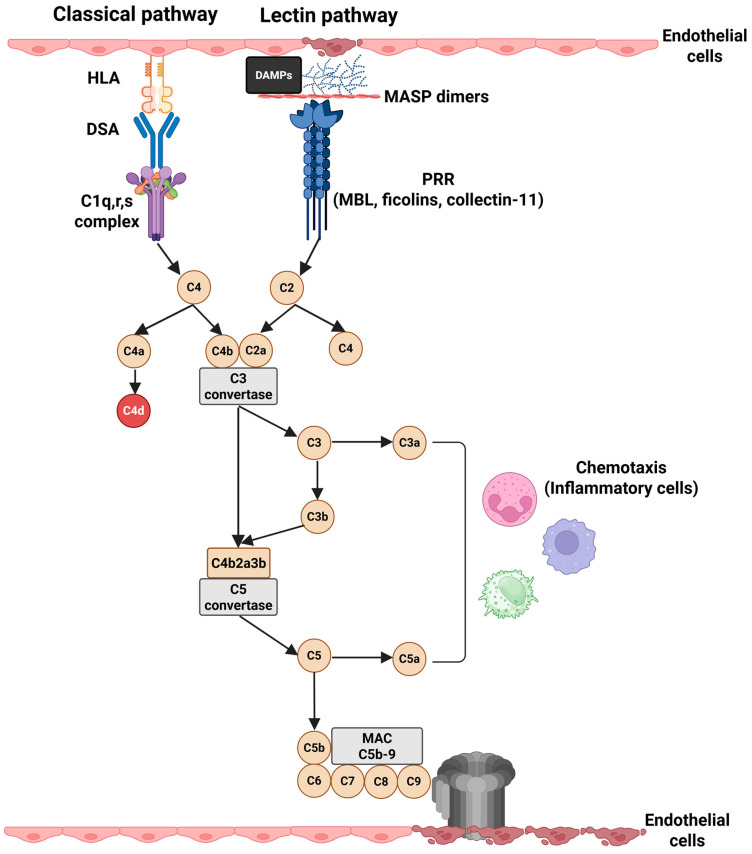
Classical and lectin complement pathways activation in AMRs (HLAs—human leukocyte antibodies; DSAs—donor-specific antibodies; DAMPs—damage-associated molecular patterns; MASP—mannan-binding lectin serine protease; PRR—pattern recognition receptor; MBL—mannose-binding lectin; MAC—membrane attack complex).

Kidney transplantation (KT) is the optimal therapeutic method for patients with end-stage kidney disease (ESKD) [1]. Antibody-mediated rejection (AMR) remains the main immunological complication after KT and a leading cause of kidney graft loss [2,3]. Its incidence can exceed 30% in the first 5 years after KT [2,4]. The presence of preformed or de novo donor-specific antibodies (DSAs) against the human leukocyte antigen (HLA) has been regarded as the main cause of AMR [5]. They can mediate kidney graft injury primarily in a complement-dependent manner [6]. More recently, several antibodies against non-HLA (non-HLA Abs) have been shown to induce graft injury either by complement-dependent or -independent mechanisms [7,8]. The complement system is an essential component of the innate immune system. Its role in KT has been described in various scenarios, including ischemia–reperfusion injury (IRI), AMR, complement-mediated recurrent disease (atypical hemolytic uremic syndrome, C3 glomerulopathy), and in the accommodation phenomenon from ABO-incompatible KT [9]. The role of complement in the pathophysiology of AMR has been extensively studied [6]. The classical complement pathway is majorly involved, but activation of the lectin pathway is also important in the pathophysiology of active and chronic AMR (Figure 1) [10].

Despite the advances in the understanding of pathophysiology and diagnosis of AMR, its treatment remains a major challenge. Current recommendations for AMR management are largely based on low-level evidence from observational studies and expert opinion [11]. Given the significant role of complement activation in AMR, terminal and proximal complement blockade has gained recognition in the management of AMR [12,13]. Several novel complement inhibitors have been tested or are under investigation in clinical trials for other diseases and could also be promising for AMR management.

The purpose of this narrative review is to provide the current understanding of the main pathophysiological events related to complement-mediated kidney graft injury in AMR, to present the current evidence regarding complement blockade in AMR management, and to point out the emerging therapies and future directions in this area.

## 2. Complement System in Kidney Transplantation

The complement system is an essential element of innate immunity and is involved in kidney graft injury [14]. In the setting of KT, complement activation could occur at many steps during the transplantation process, beginning with the manipulation of the graft before implantation, continuing with IRI, and finishing with graft failure [9]. Activation has been demonstrated to arise in any of the three pathways: the classical pathway (CP), lectin pathway (LP), and alternative pathway (AP) [14]. The presence of some factors related to the recipient, donor or transplant could favor increased complement activity in the transplanted kidney (Table 1) [9,15,16,17].

Some evidence suggests that complement activation is present in the kidneys of brain-death and circulatory-death donors prior to implantation [18]. In brain-death donor kidneys, complement activation via AP occurs both locally and systemically, contributing to inflammation and graft injury after implantation [19]. Elevated levels of C3a, C5a, and C5b-9 have been detected in the sera of deceased donors [20]. In addition, immunofixation for C3 has been observed in vascular endothelial cells and the glomerular area of brain-death donor kidneys [19].

IRI is unavoidable in KT. This process involves a two-stage injury, one determined by ischemia and the other by reperfusion. During ischemia, the kidney is subjected to hypoxia, which results in an acidic environment and a switch to anaerobic cell metabolism. This results in adenosine triphosphate (ATP) depletion, release of damage-associated molecular patterns (DAMPs), and architectural damage to endothelial and renal tubular cells. During reperfusion, cellular damage is exacerbated by the generation of reactive oxygen species. Damage of endothelial cells decreases surface complement regulation, favors complement activation, and promotes an inflammatory environment that sustains the process. Preclinical and clinical studies have shown that during IRI, complement activation occurs through the AP and LP and that the main effectors of kidney graft injury are C3a, C5a, and C5b-9 [21]. Prolonged cold ischemia time is consistent with the extent of IRI, complement activation, and graft failure [22]. From a practical point of view, this information is important because it could impact the therapeutic approach of patients with prolonged CIT, by using normothermic perfusion machines or even prophylactic use of complement inhibitors to prevent its activation and subsequent lesion occurrence and to improve graft survival.

Chronic allograft injury can have both immune and nonimmune causes. The targets of injury include the glomerular and peritubular capillaries endothelium, as well as the tubular epithelium [23]. In response to chronic injury, the affected structures respond by tissue remodeling and repair processes, which leads to interstitial fibrosis and tubular atrophy, glomerulosclerosis, thickening of the glomerular basement membrane, and vascular lumens [24]. This is associated with kidney graft function decline and failure [24]. The involvement of complement activation in interstitial fibrosis and tubular atrophy development has been documented in basic research. There is evidence that complement activation occurs through the LP via collectin-11 [25]. In addition, increased expression of C1r, C1s, C3, and the C5a/C5aR1 axis in the kidney may play an important role [26,27,28].

After transplantation, the relationship between infections, underimmunosuppression, and complement activation in KT recipients is of interest. Infections such as cytomegalovirus or BK virus can trigger complement activation and promote the development of DSA, which can culminate in AMR. Underimmunosuppression due to non-compliance or even a reduction in immunosuppression, in the context of severe infections, could lead to DSA formation, complement activation, and an increase in the risk of AMR. The judicious management of immunosuppression and infections in an individualized manner is decisive for preventing complement-mediated graft injury [29,30,31].

## 3. The Role of Complement in the Pathophysiology of AMR

The vascular endothelium is the interface between the kidney graft and the recipient blood. It is also the site of antibody–antigen interactions in AMR [32]. DSA against HLA (anti-HLA DSA) can cause antibody-mediated injury through complement-independent and complement-dependent mechanisms [33]. Complement-independent mechanisms involve direct injury of the capillary endothelium produced by DSA or indirect injury by the recruitment of inflammatory cells. These mechanisms may be encountered more frequently in AMR with non-HLA antibodies [32,34]. Complement-dependent mechanisms require the presence of DSA with fixing-complement properties against HLA or non-HLA, the formation of immune complexes on the surface of the endothelium and complement activation by the CP (Figure 1). This mechanism is seen in AMR with anti-HLA antibodies [32,34].

In the AMR process, anti-HLA DSA can be either preformed or de novo. Apart from their capacity to bind complement, their specificity, strength, and IgG subclass can also modulate the AMR phenotype [33]. DSA with higher mean fluorescence (MFI) intensity and increased capacity for binding C1q and C3d, belonging to the IgG3 subclass, are highly pathogenic and are associated with negative graft outcomes [6]. The role of complement in AMR pathophysiology begins when immune complexes bind circulating C1q. Subsequently, serine proteases C1r and C1s are activated and become capable of cleaving C4 into C4a and C4b fragments [6]. C4a can activate protease-activated receptors 1 and 4, leading to stress fiber formation in endothelial cells and increased endothelial permeability [14]. C4b on the one hand participates in the formation of the C3 convertase of CP and is subsequently cleaved to form C4d, an important histopathological feature of AMR [15]. During complement activation, anaphylatoxins (C3a and C5a), C3b, and C5b-9 are generated [14]. C3a and C5a determine the production of chemotactic cytokines and chemokines (interleukin-1, interleukin-6, interleukin-8, chemokine C-C motif ligand 5) and increase the expression of adhesion molecules (vascular cell adhesion protein-1, intercellular adhesion molecule-1, E-selectin) [34]. The proinflammatory molecules mentioned above favor the migration and adhesion of leukocytes. This process is crucial for the appearance of glomerulitis and peritubular capillaritis, which define microvascular inflammation (MVI) in AMR [34,35]. One of the roles of C3b is to opsonize antigens. Thus, the C3-opsonized antigen bound to complement receptor 2 from follicular dendritic cells promotes B-cell activation, antibody production, and amplification of the adaptive immune response [36,37,38]. In addition, it promotes the binding of polymorphonuclear leukocytes and monocytes to the endothelial cell surface [39]. Finally, C3b mediates and amplifies complement activation via AP [40]. C5b-9 can directly induce sublethal injury to endothelial cells or indirectly modify their function. C5b-9 may induce endothelial cell proliferation and generate a proinflammatory and procoagulant environment that contributes to the formation of MVI and thrombosis [34,40].

AMR is a continuous process with varying degrees of active and chronic lesions. AMR often evolves as a chronic progressive process, and it has been proven that insidious preformed or de novo DSA are involved in late onset AMR with chronic features [11]. Longstanding activity of DSA and persistence of MVI create a pattern of repeated injury on the vascular endothelium, which leads to the occurrence of chronic lesions such as transplant glomerulopathy or peritubular capillary basement membrane multilayering [41]. As in the case of active histological lesions, complement is also involved in the development of chronic lesions [42]. In a study of 205 patients with preformed and de novo DSA, those with de novo DSA had more chronic lesions of AMR, similar C4d positivity in peritubular capillaries, and inferior graft survival than patients with preformed DSA [5].

## 4. Histological Features of AMR and Complement

AMR is a clinicopathological entity whose diagnosis is based on morphological, molecular, and serologic criteria according to the Banff classification [43,44]. Depending on the presence of active and chronic histological lesions, there are three subtypes of AMR: active AMR, chronic active AMR, and chronic AMR [43].

The three mandatory criteria for the diagnosis of active AMR are histological evidence of acute tissue injury, evidence of current/recent antibody interaction with the vascular endothelium, and serologic evidence of circulating DSA against HLA and non-HLA. The first criterion could be fulfilled by at least one of the following: MVI, intimal or transmural arteritis, or acute thrombotic microangiopathy (TMA) [43]. After the Banff 2022 Kidney Meeting Report, acute tubular injury was removed from the list of key histological features of AMR. It has been considered that its presence as a solitary feature is too common and remains to be reported along with other AMR lesions to further support the diagnosis of AMR [44]. The second criterion could be fulfilled by at least one of the following: linear C4d staining in peritubular capillaries or medullary vasa recta, at least moderate MVI, and increased expression in the biopsy tissue of gene transcripts/classifiers strongly associated with AMR. The last criterion could be satisfied by demonstrating DSA against HLA or non-HLA in the sera of patients. Linear staining for C4d or expression of gene transcripts may substitute DSA in this last criterion [43].

Chronic active AMR is a histopathological pattern that can encompass lesions with severe activity and mild chronicity or those with mild activity and severe chronicity or intermediate cases. The three mandatory criteria for the diagnosis of chronic active AMR are morphological evidence of chronic tissue injury, evidence of current/recent antibody interaction with the vascular endothelium, and serologic evidence of circulating DSA to HLA and non-HLA. The first criterion is fulfilled by the presence of transplant glomerulopathy or severe peritubular capillary basement membrane multilayering [43]. After the Banff 2022 Kidney Meeting Report, arterial intimal fibrosis of new onset, as a solitary lesion, was removed from the diagnostic features of AMR due to its nonspecific status [44]. The second and third criteria were identical to those for active AMR [43].

Frequently, AMR has a chronic progressive evolution, and its diagnosis could be confirmed in an advanced stage of chronicity without any active lesions [11]. Chronic AMR diagnosis is fulfilled based on three mandatory criteria: presence of transplant glomerulopathy or severe peritubular capillary basement membrane multilayering, absence of evidence of current/recent antibody interaction with the vascular endothelium, and prior documented diagnosis of AMR and/or documented evidence of DSA [43].

Feutch et al. demonstrated for the first time the link between AMR and complement by identifying the presence of complement cleavage product C4d in the peritubular capillaries of kidney graft biopsies [45]. Collins et al. subsequently confirmed that C4d deposits in peritubular capillaries in the presence of DSA are markers of AMR. In addition, the authors hypothesized that the endothelium of peritubular capillaries is the major site of complement activation because there are fewer anti-complement protective pathways than the endothelium of glomerular capillaries [46]. Activation of the complement system is triggered by DSA and plays a critical role in the occurrence of both active and chronic lesions from antibody-mediated injury, such as glomerulitis, peritubular capillaritis, TMA, transplant glomerulopathy, and peritubular capillary basement membrane multilayering [47]. Nevertheless, in a study of 157 protocol biopsies from 80 patients with DSA, all C4d-positive biopsies and more than 50% of C4d-negative biopsies presented MVI, which could indicate that complement activation is not always mandatory for inflammation development and antibody-mediated injury [48].

C4d is a complement split product resulting from CP or LP activation and is considered a footprint of antibody–antigen interaction on the surface of endothelial cells [49]. C4d’s structure allows it to form strong and durable covalent bonds with the vascular endothelium [49]. C4d detection in the biopsy specimens could be performed by either immunofluorescence on frozen tissue or by immunohistochemistry on paraffin-embedded tissue. By immunofluorescence, Cd4 staining should be focal (−10–50% of peritubular capillaries and medullary vasa recta) or diffuse (>50% of peritubular capillaries and medullary vasa recta) in a linear pattern to be considered a criterion for current/recent antibody interaction with the vascular endothelium or an equivalent for serologic criterion (DSA). Using immunohistochemistry, minimal staining for C4d (>0% but <10% of peritubular capillaries and medullary vasa recta) is sufficient to fulfill the aforementioned criteria [50]. Although C4d was initially thought to be a universal marker for AMR diagnosis, it was later shown to have limitations and low sensitivity. The main identified limitations were methodological issues (interpretation, staining, technique), poor understanding of the meaning of minimal or focal staining, low sensitivity for chronic AMR diagnosis, influence of C4d expression depending on peritubular capillaries density, positivity of Cd4 in the absence of other graft lesions, and lack of usefulness in the context of non-HLA antibodies [35]. Thus, the concept of a C4d-negative AMR has emerged. Several studies have shown that lesions of MVI are associated with other acute or chronic lesions and with the presence of DSA, even in the absence of C4d. Moreover, some studies have shown that high endothelial activation and injury transcripts or certain gene expression are associated with histologic lesions of AMR and DSA, in the absence of C4d [48,51,52,53,54]. Currently, C4d positivity is no longer a mandatory feature for AMR diagnosis and can be replaced by the presence of MVI or by molecular diagnosis of gene transcripts/classifiers [43]. However, an independent association between C4d and MVI with acute and chronic forms of AMR as well as with negative outcomes has been clearly demonstrated [35]. Thus, the two histologic lesions together with DSA are the mainstay in differentiating between AMR, probable AMR, and entity of MVI, DSA-negative, C4d-negative, according to the Banff 2022 classification [44].

## 5. Complement Inhibition in the Treatment of AMR

Treatment of AMR remains a major challenge. Current recommendations for AMR management are largely based on low-level evidence from observational studies and expert opinions. Strategies have been addressed to preserve renal function, improve/reduce histologic lesions, and decrease antibody load depending on the clinical-histologic phenotypes of rejection [11]. AMR treatment includes a combination of different interventions aimed at combating pathophysiological events, including therapeutic plasma exchange, intravenous immune globulins, anti-CD20 monoclonal antibodies, proteasome inhibitors, interleukin-6 inhibitors, and imlifidase [11]. Unfortunately, these therapeutic regimens are not always effective, and sometimes evolution leads to persistence or recurrence of AMR and ultimately to chronic lesions and graft loss [11]. Given the significant role of complement activation in AMR, terminal and proximal complement blockades have gained recognition in the treatment and prevention of AMR.

### 5.1. Clinical Evidence for Terminal Complement Blockade in AMR

Several studies have evaluated the role of eculizumab as an inhibitor of the terminal complement pathway in the prevention and treatment of AMR. Eculizumab is a humanized IgG_2/4k_ monoclonal antibody that binds to the C5 component of the complement system and blocks its cleavage by C5 convertase in C5a and C5b. In this manner, it prevents the proinflammatory, prothrombotic, and lytic effects of C5b-9. This drug is approved for the treatment of paroxysmal nocturnal hemoglobinuria and atypical hemolytic uremic syndrome.

Regarding its role in the prevention of AMR, observational studies have suggested a beneficial contribution, but no advantages in terms of patient and graft survival [55,56,57]. Data from a phase 2, open-label, randomized controlled trial (RCT) showed that eculizumab had no effect on preventing graft failure or grade II/III AMR episodes compared with standard of care (SOC) [58]. However, a benefit was observed when the definition included grade I-III AMR [58].

Regarding its role in the treatment of AMR, evidence comes from case reports, case series, observational studies, and two RCTs (Table 2) [12,59,60,61,62,63,64,65,66,67,68,69,70,71,72,73,74]. Most studies included adult kidney transplant recipients; however, there are also case reports of pediatric patients [63,65]. Most patients included in the reported studies were ABO-incompatible, HLA-incompatible, or sensitized. Eculizumab has been used in the treatment of both active and chronic forms of AMR [12,64,65,66,74]. Active AMR treatment has been reported for both the early- and late-onset forms [64,65]. In a retrospective observational study of 15 sensitized KT recipients with early active AMR, with a mean diagnostic time of 10 days (IQR: 7–11), treatment with eculizumab added to therapeutic plasma exchange (80%) or splenectomy (6.7%) has been shown to improve kidney graft function and histological lesions of AMR [64]. The median estimated glomerular filtration rate (eGFR) increased from 21 to 45 mL/min after 12 months of follow-up, the histological evidence of AMR on surveillance biopsies decreased by 83.3%, and there were no graft losses [64]. In a case report study of two KT recipients with late-onset acute AMR, eculizumab was added to plasma exchange, intravenous immunoglobulin, and rituximab-induced recovery of kidney function and avoided graft loss [65]. The effect of eculizumab as a therapeutic modality for chronic AMR was evaluated in a pilot RCT. The sample size included 15 patients with de novo DSA, mostly C4d-negative AMR (80%). Among them, ten were randomized to the eculizumab group and five to the control (observational) group. After six months of treatment, it was concluded that the eGFR trajectory was stable in the eculizumab group, but without significance when compared with the non-interventional group. In addition, no significant reduction in endothelial-associated transcript (ENDAT) expression was observed after eculizumab treatment in chronic AMR [12]. These findings highlight that endothelial injury in AMR is not complement-mediated in all cases and that the use of eculizumab in chronic AMR may attenuate lesions and stabilize graft function. A case report showed that eculizumab used in two KT recipients with C4d-negative AMR did not lead to recovery of kidney graft function and resulted in graft loss in both cases [73]. Eculizumab has also been tested for refractory AMR. Two studies, one case report and a case series including 10 patients with AMR refractory to other therapies, showed that eculizumab use was associated with a 50% graft survival [61,71]. The only randomized clinical trial published to date that evaluated the treatment of AMR with eculizumab was conducted in 11 patients with active and chronic active forms of AMR [66]. Among them, seven patients received eculizumab and four received SOC (therapeutic plasma exchange/intravenous immunoglobulins). More than 80% of patients were sensitized, the median time of AMR diagnosis was 1628 days (range: 1–5495), and all biopsy specimens were C4d-positive. The results of this RCT showed that treatment with eculizumab alone is not sufficient for the treatment of AMR or for the prevention of acute AMR to chronic AMR progression or transplant glomerulopathy. One important limitation in reaching the endpoint was the fact that most patients had chronic AMR lesions at the time of treatment initiation [66]. In a retrospective study that also included patients with active and chronic AMR, the combination of eculizumab plus splenectomy combined with SOC treatment showed a significant reduction in transplant glomerulopathy occurrence and was the only factor associated with 100% graft survival compared to patients treated with eculizumab alone or splenectomy alone [74]. An important aspect in clinical practice is related to C4d immunofixation. Although it is not mandatory for the diagnosis of AMR, when it is positive in peritubular capillaries, it highlights a complement-dependent mechanism involved in the occurrence of rejection lesions and can be a criterion for the use of anti-complement therapy.

Current evidence regarding the use of eculizumab for the treatment of AMR is weak and does not support its routine use. Evidence is limited due to the small number of patients, study designs, significant heterogeneity in the populations studied, and the treatment regimens used. Although eculizumab remains an option for the treatment of AMR, it should be considered alongside other therapies such as therapeutic plasma exchange and intravenous immunoglobulin ± rituximab in patients with active or chronic active AMR and not used as a solitary therapy [11]. The doses of eculizumab vary among studies, and it is difficult to standardize them for atypical hemolytic uremic syndromes. Variability is related to availability, type of rejection, and response to treatment; therefore, an individualized approach seems explicable. For example, in case reports, the doses administered ranged from one to eight [59,67]. Orandi et al. reported a median of 12.5 doses in their study [74]. In the two RCTs mentioned above, the following regimens were used: 600 mg/week for 4 weeks and 900 mg/2 weeks for 6 months; 1200 mg first dose, then 900 mg weekly for four doses, then 1200 mg in week 5, and in week 6, if DSA < 50% of baseline DSA, then no further treatment; otherwise, 1200 mg in weeks 7 and 9 [12,66]. Treatment duration is also an important matter of debate. Based on the current evidence, a limited duration of administration may be recommended, as eculizumab does not appear to prevent chronic AMR. Furthermore, discontinuation of the treatment can be considered when DSA < 5000 MFI and the prozone is no longer present [64].

### 5.2. Clinical Evidence for Proximal Complement Blockade in AMR

Therapeutic interventions targeting proximal complement blockade in AMR treatment are also of interest. Such an approach may be possible by inhibiting the C1 component with C1 esterase inhibitors (C1-INHs) or by inhibiting C1s with monoclonal antibodies (sutimlimab) (Table 3) [13,75,76]. Theoretically, blocking CP at the C1 level inhibits complement activation triggered by immune complexes (DSA-Ag) and its role in antibody-mediated injury.

C1-INH is a serine protease inhibitor that primarily blocks CP by inhibiting C1r and C1s, and LP by inhibiting mannan-binding lectin serine protease 1 (MASP-1). In addition, it inhibits coagulation and kinin systems involved in vascular permeability and inflammation. C1-INH can also directly block leukocyte–endothelial cell adhesion [77]. Similar to eculizumab, C1-INH has been evaluated for both the prevention and treatment of AMR. In an RCT, Vo et al. evaluated the effect of C1-INH compared to placebo on AMR occurrence [78]. Twenty highly sensitized KT recipients were enrolled, ten in the intervention arm and ten in the placebo arm. The C1-INH dose was 20 units/kg/dose intraoperatively and then twice weekly for seven doses. During the study, no C1-INH patients developed AMR compared with one patient in the placebo arm [78]. Viglietti and colleagues investigated in a single-arm, prospective, pilot study the effects and safety of C1-INH added to high dose intravenous immunoglobulin for the treatment of unresponsive active AMR. After 6 months of treatment, authors observed a significant improvement of eGFR from 38.7 ± 17.9 to 45.2 ± 21.3 mL/min, a decrease in C4d deposition rate from 5/6 to 1/6 and DSA C1q status, with a good safety profile [13]. A phase 2b, multicenter, double-blind RCT by Montgomery et al. evaluated the effect of plasma-derived C1-INH added to standard of care (SOC) in AMR treatment. Eighteen patients were enrolled and randomized, among whom nine received 20.000 U of C1-INH divided into seven doses over a 2-week period, and nine received placebo. The results showed that C1-INH was well tolerated, and no graft survival difference was reported after 20 days. Six-month surveillance biopsies revealed 0% transplant glomerulopathy in the C1-INH group and 42.8% in the placebo group. Another important finding was that therapeutic plasma exchange significantly depleted endogenous C1-INH [75].

Sutimlimab, previously known as TNT009 or BIVV009, is a monoclonal antibody against C1s that effectively blocks CP [79]. In phase 1b, a non-randomized trial of ten KT recipients with late active or chronic AMR and features of CP activation, four weekly doses of sutimlimab (60 mg/kg) were well tolerated and led to a sustained blockade of CP in the serum and kidney tissue but had no significant impact on kidney function, DSA levels, MVI, and gene expression [76].

Studies on the treatment of AMR with C1 inhibitors are limited by small sample sizes and cannot lead to solid conclusions. However, based on the existing data, this could be an option when added to the SOC. They are generally well tolerated, effectively block C4, may improve graft function, and may prevent progression to chronic AMR. Nonetheless, more randomized studies with larger numbers of patients are needed to better characterize the target patient group that would benefit more from the treatment.

## 6. Novel Therapies and Future Directions

Several novel complement inhibitors have been tested or are under evaluation in clinical trials for other diseases and could also be promising for AMR management. In addition, encouraging data were obtained from preclinical studies and xenotransplantation studies.

Ravulizumab is a humanized IgG_2/4k_ monoclonal antibody against C5 that targets the same epitope as eculizumab, but with long-lasting action and a reduced schedule of administration. To date, KT has only been used for the treatment of atypical hemolytic uremic syndrome [80,81]. Its potential use for the treatment of AMR seems promising, but it remains to be seen whether the long duration of action will be an impediment given that C5 inhibitors are typically used for a short, limited duration in AMR.

Tesidolumab (LFG316) is a recombinant, high-affinity, human monoclonal antibody of the IgG1/lambda isotype, directed against human C5 [82]. This was evaluated in a pig-to-rhesus kidney xenotransplantation model to explore the possibility of minimizing early acute AMR in the context of costimulatory blockade-based immunosuppression. Temporary use of tesidolumab reduced early graft loss secondary to AMR and improved graft survival [83].

Avacopan is a C5a receptor 1 inhibitor used in the treatment of ANCA-associated vasculitis and is under investigation for C3 glomerulopathy treatment. Limited data have been reported on IgA nephropathy treatment [84]. Avacopan could offer potential benefits in the treatment of AMR by reducing complement-mediated inflammation and tissue damage. However, targeting only the inflammatory loop may be insufficient and may require combination with other therapies.

Nomacopan is a second-generation complement inhibitor that targets C5 independently and leukotriene B4, both of which are co-located as part of the immune/inflammatory response. The drug is under investigation in an open-label study of patients with hematopoietic stem cell transplant-associated TMA (NCT04784455). Given that its mechanism of action is similar to eculizumab and its additional anti-inflammatory properties, it could be a promising solution for the treatment of AMR. Other C5 inhibitors under investigation are cemdisiran and gefurulimab [84].

C5 Small-interfering RNA Lipid Nanoparticle (C5 siRNA-LNP) was investigated in a basic research study for its effect on the downregulation of complement activity and AMR treatment. This novel therapeutic approach uses siRNA, which directly reduced mRNA levels of C5. C5 siRNA-LNP completely suppressed C5 expression and complement activity, effectively attenuating AMR and prolonging graft survival in this rat model [85].

Compstatin (Cp40) is a cyclic peptide that blocks convertase-mediated activation of C3 via all complement pathways. In a non-human primate model, Cp40 prevented early AMR and prolonged graft survival in the presence of high levels of DSA, and showed immunomodulatory effects [86].

Pegcetacoplan is a pegylated peptide that targets complement C3 and inhibits its activity. It also binds to and prevents the activity of C3b, thus inhibiting the activity of C3 and C5 convertases of AP and C5 convertase associated with CP. This new molecule has sparked interest in the KT field for C3 glomerulopathy recurrence treatment and has demonstrated its efficacy, safety, and tolerability in a prospective, phase 2, multicenter, open-label, randomized controlled trial [87]. Given its mechanism of action, it is also emerging as a molecule of interest for AMR treatment.

Novel therapies for blocking LP have been developed in the field of kidney disease. Considering the involvement of LP in AMR, these new molecules are of great interest. Narsoplimab is a human monoclonal antibody against MASP-2 that has been investigated for the treatment of patients with IgA nephropathy (NCT03608033).

Evidently, there is an abundance of new complement inhibitors. As research progresses, integrating these novel therapies into AMR management could lead to more effective and tailored approaches.

## 7. Conclusions

Complement plays a crucial role in the entire transplant process, particularly in the onset and progression of AMR. Although remarkable progress has been made in understanding the pathophysiology of AMR, this has not yet been fully elucidated. There is a balance between complement-dependent and -independent mechanisms in the development of rejection lesions, which vary from patient to patient. Understanding the pathophysiological stages is fundamental for therapeutic approaches. Current evidence regarding AMR treatment is limited and of a low quality. The use of complement inhibitors in active and chronic active AMR, whether targeting the proximal or terminal pathways, should be part of a personalized approach as an adjunctive therapy to SOC. To better characterize and select patients who would respond to complement inhibitors, future RCTs with larger sample sizes are needed, along with improved risk stratification based on DSA and complement features, better standardization of histological lesions, and use of novel molecular and genetic markers. The ongoing investigation of novel complement inhibitors highlights a significant shift towards a more precise approach against AMR. Continued research and development in xenotransplantation will provide further insight into the management of complement-mediated AMR.

## Figures and Tables

**Table 1:** Potential factors prior to or during KT that may increase complement activity in the transplanted kidney.

Recipient-Related Factors	Donor-Related Factors	Transplant-Related Factors
***Comorbidities*** Diabetes HTN Smoking	***Donor type*** Cadaveric donors (DBD, DCD)	***Graft preservation*** Cold storage Normothermic machine perfusion
***Cause of CKD*** Atypical hemolytic uremic syndrome C3 glomerulopathy Lupus nephritis AAV	***Comorbidities*** HTN	***Ischemia times*** Increased WIT Increased CIT
***Type of dialysis*** Hemodialysis		***Immunosuppression*** CNI toxicity

HTN—hypertension; CKD—chronic kidney disease; AAV—ANCA-associated vasculitis; WIT—warm ischemia time; CIT—cold ischemia time; DBD—donation after brain death; DCD—donation after circulatory death; CNI—calcineurin inhibitors.

**Table 2:** Summary of studies assessing treatment with eculizumab in AMR.

Author (Year)	Study Type	Patients No.	Transplant Type	AMR Time (Post-KT)	AMR Type	Eculizumab No. of Doses	Other Treatments	Response	Graft Loss
Locke et al. (2009) [59]	Case report	1	HLAi	D10	Active AMR, C4d+	1	PE, IVIG, anti-CD20	Yes	No
Biglarnia et al. (2011) [67]	Case report	1	ABOi	D9	Active AMR, C4d+	2	IA	Yes	No
Stewart et al. (2012) [68]	Case report	1	ABOi	D4	Refractory active AMR, C4d+	8	PE, IVIG, splenectomy	Yes (partially)	No
Gonzalez-Roncero et al. (2012) [69]	Case report	2	1st-Retransplant, HLAi; 2nd-first KT;	1st-D7; 2nd-D8;	1st-Active AMR, vascular thrombosis, C4d+; 2nd-Active AMR, TMA, C4d+;	1st-1; 2nd-1;	1st-PE, IVIG, anti-CD20 2nd-PE, IVIG, anti-CD20;	1st-Yes; 2nd-Yes;	1st-No; 2nd-No;
Noone et al. (2012) [70]	Case report	1	Retransplant (highly sensitized)	D15	Refractory chronic active AMR, TMA, C4d+	2	PE, IVIG, anti-CD20	Yes (partially)	No
Kocak et al. (2013) [71]	Case report	2	1st-Retransplant (sensitized); 2nd-First KT (sensitized);	1st-D8; 2nd-D5;	1st-Active AMR, TMA, severe vascular lesions, C4d+; 2nd-Mixed acute rejection, C4d+;	1st-5; 2nd-5;	1st-PE, IVIG, anti-CD20; 2nd-IVIG, anti-CD20;	1st-Yes; 2nd-No;	1st-No; 2nd-Yes;
Ghirardo et al. (2014) [72]	Case report	1	Retransplant (highly sensitized)	D42	Active AMR, C4d+	4	PE, IVIG, anti-CD20	Yes; 1 year graft biopsy showed complete resolution of AMR;	No
Burbach et al. (2014) [73]	Case report	2	1st-First KT (sensitized); 2nd-Retransplant (sensitized);	1st-12 months; 2nd-1 month;	1st-Chronic active AMR, C4d−; 2nd-Active AMR, C4d−;	1st-N/A; 2nd-15;	1st-PE, IVIG, anti-CD20; 2nd-IA, IVIG;	1st-No; 2nd-Yes (temporary);	1st-Yes 2nd-Yes
Orandi et al. (2014) [74]	Retrospective	24 (14/24-Sple-nectomy; 5/24-Eculi-zumab+ splenectomy; 5/24-Eculi-zumab)	HLA/ABOi	Median: 6 days (5–9)	Active and chronic AMR, C4d+	Median: 12.5 (4–17)	PE, IVIG, anti-CD20	Similar mean SCr across the groups at 1-year after KT; Transplant glomerulopathy at follow-up biopsies: 70% (Splenectomy group), 20% (Eculizumab+ splenectomy group), 100% (Eculizumab group);	22.1—Splenectomy group; 0—Eculizumab + splenectomy group; 70—Eculizumab group;
Chehade et al. (2015) [60]	Case report	1	Retransplant (highly sensitized)	D4	Active AMR, C4d+, borderline TCMR;	2	PE, IVIG, ATG	Yes; 2 months graft biopsy showed complete resolution of AMR;	No
Yelken et al. (2015) [61]	Case series	8	2/8-Retransplant; 4/8-Sensitized patients;	3/8-first 3 days; 5/8-between 8 and 18 months;	5/8-Acute AMR 6/8-C4d+ 1/8-TMA	N/A	PE, IVIG, RTX, ATG,	4/8-Yes	4/8-Yes
Smith et al. (2016) [62]	Case report	2	ABOi	1st-D7; 2nd-D5;	1st-Active AMR, C4d+; 2nd-Active AMR, C4d+;	1st-2; 2nd-4;	PE, IVIG, anti-CD20, splenectomy;	1st-Yes; 1 year graft biopsy showed complete resolution of AMR; 2nd-Yes (partially); 1 month biopsy showed partial resolution of the histological lesions of AMR;	1st-No; 2nd-No;
Lanfranco et al. (2017) [63]	Case report	2	ABOi	1st-D13; 2nd-D12	1st-Active AMR, C4d+, TMA, vascular thrombosis; 2nd-Acute AMR, Cd4+, TMA;	1st-6; 2nd-6;	1st-PE, IA; 2nd-PE, IA;	1st-No; 2nd-Yes;	1st-Yes; 2nd-No;
Kulkarni et al. (2017) [12]	Pilot RCT	15 (Treatment group: 10 patients—6 months of Eculizumab and 6 months of observation; Control group: 5 patients—observation only)	Chronic AMR, de novo DSA MFI > 1100,	N/A	Chronic AMR, C4d+ (20%)	14	N/A	Stable graft function and non-significant mean eGFR difference between groups at 6 months:−1.52 (8.80 to 5.76)	No reduction in ENDAT with complement inhibition on protocol biopsies at 1 year
Tan et al. (2019) [64]	Retrospective	15	Sensitized patients (80%) + B-FCXM (66%)	Median: 10days (7–11)	Early active AMR, C4d+	Median: 5	PE (80%), splenectomy (6.7%)	Improvement of kidney function; AMR resolution (83.3%) within 4–6 months;	No
Siddiqui et al. (2022) [65]	Case report	2	Unsensitized patients	1st-11 months; 2nd-24months;	Late active AMR, C4d+	1st-2; 2nd-2;	1st-PE, IVIG, anti-CD20; 2nd-PE, IVIG, anti-CD20;	1st-Yes; 2nd-Yes;	1st-No; 2nd-No;
Heo et al. (2022) [66]	RCT, open label	11 (Eculizumab arm: 7 patients; SOC arm: 4 patients)	Sensitized patients (81.8%); Retransplant (27.3%)	Median: 1628 days (1–5495)	Active and chronic active AMR, C4d+	7 (if DSA < 50% of baseline DSA; 9 (if DSA > 50% of baseline);	PE, IVIG, anti-CD20, bortezomib, ATG	Lack of efficacy in AMR treatment and in prevention of acute AMR to chronic AMR progression or transplant glomerulopathy	No

no.—number; KT—kidney transplant; AMR—antibody-mediated rejection; HLAi—HLA incompatible; ABOi—ABO incompatible; D—day; PE—plasma exchange; IVIG—intravenous immunoglobulins; IA—immunoadsorption; +—positive; −—negative; TMA—thrombotic microangiopathy; N/A—not available; DSA—donor-specific antibodies; MFI—mean fluorescence intensity; eGFR—estimated glomerular filtration rate; SCr—serum creatinine; ENDAT—endothelial-associated transcripts; B-FCXM—B flowcytometry crossmatch; SOC—standard of care; ATG—anti-timocyte globulin; RCT—randomized controlled trial; 1st—first case; 2nd—second case.

**Table 3:** Summary of studies assessing treatment with C1 inhibitors in AMR.

Author (Year)	Study Type	Sample Size	AMR Type	Intervention	Outcomes
Viglietti et al. (2016) [13]	Single-arm, pilot	6	Active AMR unresponsive to SOC therapy	C1-INH 2000 U/day for 3 days, then twice weekly added to high dose IVIG for 6 months;	eGFR improvement from 38.7 ± 17.9 to 45.2 ± 21.3 mL/min/1.73 m^2^ (*p* = 0.0277); No changes in histological feature, except a decrease in the C4d deposition rate from 5/6 to 1/6 (*p* = 0.0455); Change in DSA C1q status from 6/6 to 1/6 (*p* = 0.0253); AE: 1 DVT;
Montgomery et al. (2016) [75]	Phase 2b, multicenter double-blind RCT pilot study	18	Active and chronic active AMR	C1-INH arm (n = 9)—7 doses over a 2-week period, with an initial IV infusion of 5000 U on day 1, followed by 2500 U IV on days 3, 5, 7, 9, 11, and 13 added to SOC (PE, IVIG ± RTX); Placebo arm (n = 9);	Graft survival at D20 between groups was not different; Six-month biopsies (n = 14): Transplant glomerulopathy in 0/7 (C1-INH arm) and 3/7 (Placebo arm); PP significantly depleted endogenous functional C1-INH levels; No discontinuations, graft losses, deaths, or study drug—related serious AEs;
Eskandary et al. (2018) [76]	Non-randomized, phase 1b trial	10	Late active or chronic active AMR	Anti-C1s monoclonal antibody—4 weekly doses (60 mg/kg);	No serious AEs were observed during the 7 weeks of follow-up; Sutimlimab profoundly blocks CP activity in both serum and renal tissue; 5/8 C4d+ KTR switch to a C4d− status and 2/8 KTR had a significant decrease in C4d score; No change in microcirculation inflammation, gene expression patterns, DSA levels, or kidney function;

AMR—antibody-mediated rejection; SOC—standard of care; eGFR—estimated glomerular filtration rate; C1-INH—C1 esterase inhibitor; U—units; IV—intravenous; PE—plasma exchange; IVIG—intravenous immunoglobulin; RTX—rituximab; n—number; D—day; AE—adverse events; KTR—kidney transplant recipients; CP—classic pathway; DSA—donor-specific antibodies.

## Data Availability

Not applicable.

## Abbreviations

AL—alternative pathway; AMR—antibody-mediated rejection; ATP—adenosine triphosphate; C1-INH—C1 esterase inhibitor; Cp-40—compstatin; CP—classic pathway; DAMPs—damage-associated molecular patterns; DSAs—donor-specific antibodies; eGFR—estimated glomerular filtration rate; ENDATs—endothelial-associated transcripts; ESKD—end-stage kidney disease; HLA—human leucocyte antigen; IQR—interquartile range; IRI—ischemia–reperfusion injury; KT—kidney transplantation; LP—lectin pathway; Non-HLA Abs—non-HLA antibodies; MASP-1—inhibiting mannan-binding lectin serine protease; MFI—higher mean fluorescence; MVI—microvascular inflammation; RCT—randomized controlled trial; SOC—standard of care; TMA—thrombotic microangiopathy.
